# Analysis of Causative Factors and Potential Predictors of Onychomycosis: A Retrospective Single-Center Study in Poland

**DOI:** 10.3390/jof11020131

**Published:** 2025-02-09

**Authors:** Andrzej K. Jaworek, Przemysław Hałubiec, Anna Wojas-Pelc, Jacek C. Szepietowski

**Affiliations:** 1 Department of Dermatology, Jagiellonian University Medical College, Botaniczna 3, 31-503 Cracow, Poland; andrzej.jaworek@uj.edu.pl (A.K.J.); przemyslaw.halubiec@doctoral.uj.edu.pl (P.H.); anna.wojas-pelc@uj.edu.pl (A.W.-P.); 2 Doctoral School of Medical and Health Sciences, Jagiellonian University Medical College, Łazarza 16, 31-530 Cracow, Poland; 3 Department of Dermato-Venereology, 4th Military Hospital, Weigla 5, 53-114 Wroclaw, Poland; 4 Faculty of Medicine, Wroclaw University of Science and Technology, Grunwaldzki 11 sq., 51-377 Wroclaw, Poland

**Keywords:** onychomycosis, *Trichophyton rubrum*, *Candida albicans*, mycological culture

## Abstract

Onychomycosis is a fungal nail infection which has a considerable impact on the quality of life of patients. The aim of this study was to analyze onychomycosis cases with respect to fungal species, infection sites, and patient demographics such as age and sex. Furthermore, we assessed whether simple clinical and demographic data could predict positive results for mycological culture. A retrospective analysis of 2722 patients who had nail samples investigated with direct microscopy and mycological culture was performed. The fungi most frequently identified were *Trichophyton rubrum* in the toenails and *Candida albicans* in the fingernails, with a detailed incidence varying by age and sex. Predictive models, including logistic regression and k-nearest neighbors, did not provide clinically useful accuracy. Therefore, it is necessary to perform confirmatory diagnostics before starting antifungal treatment.

## 1. Introduction

Onychomycosis is a fungal infection of the nails and the most common nail disorder [1]. It leads to changes in the nail plate such as discoloration, thickening, and nail detachment [2]. Each part of the nail apparatus may be involved, including the nail plate, bed, and folds. The skin surrounding infected nails can undergo secondary changes [3]. Dermatophytes were traditionally considered the main cause of onychomycoses; however, current data indicate a significant role for yeasts and molds [4].

Toenails are the most common sites of infection due to factors such as increased humidity and temperatures, as well as pressure from footwear [5]. Dermatophytes even cause 90% of cases of onychomycosis of the toenail but only 50% of cases of fingernail onychomycosis [2]. The most prevalent dermatophytes are *Trichophyton rubrum* and *Trichophyton mentagrophytes* spp. Nondermatophyte molds such as *Aspergillus*, *Scopulariopsis*, and *Fusarium* account for up to 10% of all cases of onychomycosis [6]. *Candida albicans* is the predominant yeast which causes onychomycosis, with a particular affinity for the fingernails (up to 40% of cases). Exposure to water is an important risk factor for these infections [7].

The global prevalence of onychomycosis is estimated to be approximately 10%, representing half of all nail disorders. Detailed patterns of its primary etiological factors vary considerably by geographical region. In Europe and the United States, the prevalence ranges from 1% to 8% [8]. In some countries with particularly favorable climates (warm and humid), more than 50% of the population may suffer from onychomycosis [5]. These trends appear to be dynamic over time, necessitating continuous epidemiological surveillance.

The most common risk factors for onychomycosis include the male sex, older age (20% among individuals over 60 years of age and 50% among those over 70 years of age), coexisting skin disorders affecting the nails, and immunosuppression [9,10]. Socioeconomic factors and lifestyle also play important roles (exposure to humid environments, wearing close-fitting footwear, frequent travel, or contact with public bathing facilities) [11,12].

Onychomycosis poses a significant clinical challenge which causes functional and aesthetic disturbances, leading to psychological and psychosocial difficulties and, ultimately, to a severely altered quality of life (QoL) [13].

Standard diagnostic methods include direct microscopic examination followed by mycological culture of nail scrapings or nail clippings from suspected sites [14]. Histological examination and molecular methods, such as polymerase chain reactions (PCRs), yield lower false negative rates but are highly prone to contamination and might require multiple samples, particularly in cases involving non-dermatophyte molds [15].

Although mycological culture has extremely high specificity, its sensitivity is moderate [9]. This limitation, combined with the relatively long time required to establish a diagnosis, has led some authors to suggest the reservation of culture only in cases where identification of the causative fungi is essential for the choice of the exact medication [2]. Culture allows testing for antifungal resistance, which is critical for rational management of more difficult cases [12]. Some authors proposed redefining the “gold standard” for onychomycosis diagnosis to include a combined or stepped approach which integrates molecular testing with classical methods such as direct examination and histology [16].

A shift toward this evolving paradigm appears inevitable as new data on the limited precision of mycological culture continue to accumulate [16,17,18]. However, many centers and physicians still have limited access to large-scale molecular tests for suspected onychomycosis.

In such settings, standard culture combined with direct examination is still considered a more reliable basis for informed decision making than the empirical approach.

However, some authors have challenged this view, suggesting that empirical treatment may be safe and cost-effective [19]. In fact, many physicians in real-world practice choose to initiate empirical antifungal treatment, both topical and systemic [20]. On the other hand, this approach carries an increased risk of antifungal resistance and treatment without clinical improvement [21].

The empirical diagnosis of onychomycosis is generally based on nail morphology interpreted in the context of well-known risk factors. However, these risk factors are not considered in a structured or standardized manner, further complicating the diagnostic process.

This study aimed to identify current trends in the etiology of onychomycosis within the Polish population in Malopolska, focusing on clinical aspects such as the site of infection and the age or sex of each subject.

Furthermore, it sought to determine whether specific subgroups of patients could be identified in whom the diagnosis of onychomycosis could be made based on the clinical presentation and simple characteristics of the patient before receiving the results of the mycological culture.

## 2. Materials and Methods

The study was based on a retrospective analysis of archival data from 2722 patients with suspected onychomycosis diagnosed at the Mycological and Cytological Diagnostic Laboratory in Cracow. All samples were collected between 2017 and 2019.

The main inclusion criterion was the presence of clinical symptoms suggestive of onychomycosis, with the following acquisition of nail scrapings or nail clippings (from fingernails or toenails) for mycological culture. No restrictions regarding sex or age were applied.

The following data were extracted from the anonymized database: the sex and age of each subjects, sampling site (fingernails or toenails), and results of mycological culture. No data were available on the details of the clinical presentation, previous treatment, or coexisting symptoms of superficial fungal infections at other sites.

The samples were processed according to the guidelines set out by the Polish Dermatological Society [22]. First, the clinical material was subjected to a 10% potassium hydroxide (KOH) and 20% dimethyl sulfoxide (DMSO) solution. After that, direct microscopy was performed. Cultures in Sabouraud dextrose agar plates were kept for six weeks, and fungal species were identified based on their macro- and microscopical morphological characteristics. The lack of growth after that period was considered a negative culture result. The *T. mentagrophytes* complex includes *T. mentagrophytes*, *T. interdigitale*, and *T. benhamiae*.

The laboratory identified *Malassezia* spp. in direct microscopy after methylene blue staining.

*Candida albicans* was differentiated from other *Candida* species via the germ tube test. Briefly, the grown samples were incubated in serum at a temperature of 37 °C for 2–4 h, and then the presence of tube structures was checked [23].

This study was conducted in accordance with the Declaration of Helsinki (1975), as revised in 2013. Because this was a retrospective analysis of anonymized data received from the laboratory, institutional review board approval was waived, and individual subjects’ consent was not required.

### Statistical Analysis

Nominal data are presented as absolute counts (N) and proportions (%), while continuous data are shown as medians and interquartile ranges (Q1–Q3) due to the non-normal distribution of the data. The normality of continuous data was established using the Shapiro–Wilk test and visual evaluation of histograms. If there were missing data, then cases were excluded from analyses involving the missing variable.

A comparison of the proportions for the dichotomous variables was performed using the χ^2^ test (or Fisher’s exact test if the expected values in any cell of the contingency table were less than 5 or the total analyzed group size was less than 20).

The distribution of continuous data between dichotomous groups was compared using the Mann–Whitney *U* test due to the non-normality of the data distribution within these groups.

Receiver operating characteristic (ROC) curves were created to determine the optimal cut-off point to predict the presence of onychomycosis based on the age of the subjects using the highest Youden index.

Then, two predictive algorithms were developed to investigate whether a combination of simple demographic factors (sex and age) could allow the prediction of positive culture results from both fingernails and toenails. First, the k-nearest neighbors (KNN) method was applied, with the K value undergoing 5-fold cross-validation. The training set comprised 70% of the data, and the testing set constituted 30% (randomly selected). A confusion matrix was created to assess the accuracy of the resulting model.

Next, multivariate stepwise logistic regression models with 10-fold cross-validation with sex and age as covariables (age was used as a continuous or dichotomous variable using cut-off values determined from ROC curves). Models were compared using Nagelkerke’s pseudo-R^2^ and the Hosmer–Lemeshow test.

The acceptable probability of type I error was defined as α = 0.05.

Statistical analyses were performed using IBM SPSS Statistics for Windows version 29.0.2.0 (Armonk, NY, USA, IBM Corp).

## 3. Results

### 3.1. General Results

The results of 2722 mycological cultures were analyzed, and 771 (28%) were positive. There were more women than men (68% versus 32%). The median age of the subjects was 46 years (interquartile range: 32–59). The two most commonly identified species were *T. rubrum* (63% of all positive results) and *C. albicans* (26% of all positive results). The remaining were found in a minority of cases. Detailed group characteristics are presented in Table 1.

Four subgroups were extracted from the original data based on the location of the sampling for the mycological culture (fingernails or toenails) and its general result (negative or positive). Subjects with positive culture results at any location were statistically significantly older compared with those with negative mycological status. There was no significant age difference between subjects with onychomycosis in their fingernails and toenails. In the fingernails, the proportion of positive results was higher in women compared with men (38% versus 27%, *p* = 0.01), while in the toenails, men had a higher positive culture ratio than women (37% versus 21%, *p* < 0.001) (Table 2).

### 3.2. Distribution of Different Fungal Species Depending on the Sex and Age of the Subjects

**Figure 1:**
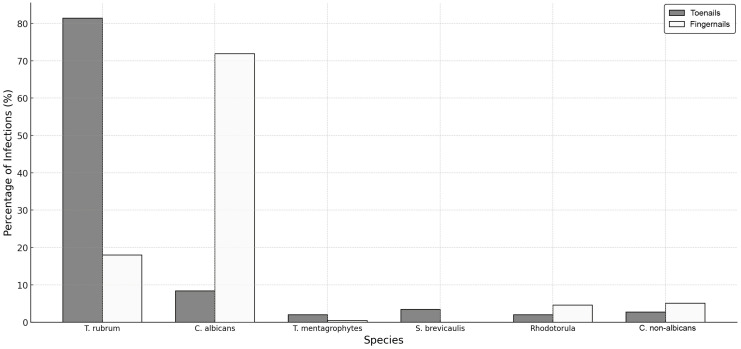
Distribution and proportion of cases identified with specific fungal pathogens by site of infection (toenails or fingernails). *T. rubrum:* 448/39 cases (*p* < 0.001); *C. albicans:* 46/156 cases (*p* < 0.001); *T. mentagrophytes:* 11/1 cases (*p* = 0.2); *S. brevicaulis:* 19/0 cases (*p* = 0.003); *Rhodotorula:* 11/10 (*p* = 0.1); *C. non-albicans:* 15/11 (*p* = 0.1). Note: *A. niger* was identified in the two cases (one in the fingernails and one in the toenails), while *M. canis* and *Malassezia* were each identified in a single case in toenails.

In the toenails, the most common cause of onychomycosis was *T. rubrum* (81.5% of all cases), while in the fingernails, *C. albicans* was the most common etiological factor of infection (71.9% of all cases). *T. rubrum* and *S. brevicaulis* were statistically significantly more frequent in toenails than in fingernails, while *C. albicans* was more common in the latter (Figure 1).

A detailed analysis of the relationship between the sex and age of the subjects with the infection site revealed that the majority of the toenail onychomycoses caused by *T. rubrum* were found in subjects between 45 and 60 years of age (both men and women). In the case of *C. albicans* fingernail onychomycosis, the highest incidence in women was the same age. However, in men, there were two smaller peaks at 15–30 and 60–75 years of age.

**Figure 2:**
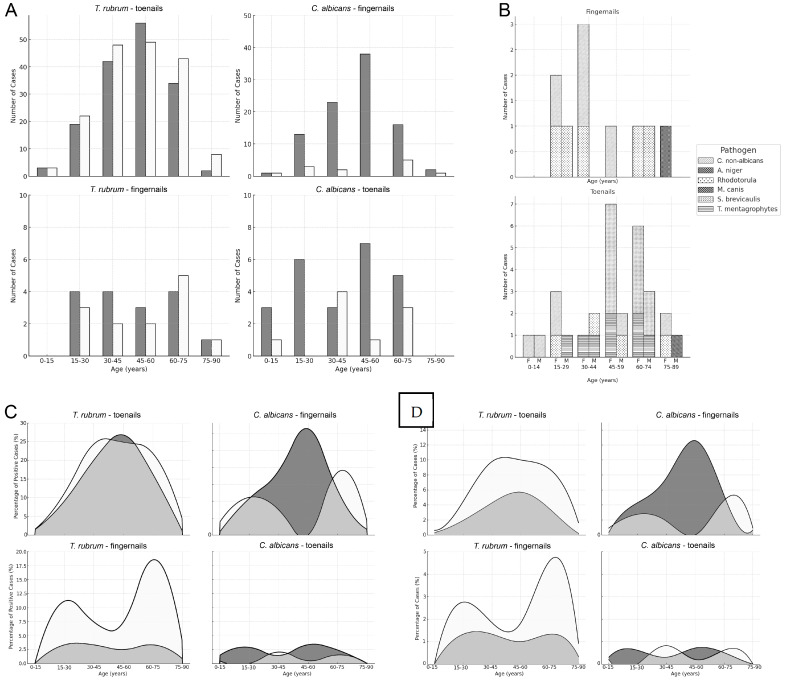
Patterns of infection by nail lesion site and subjects’ sex and age. (**A**) The absolute numbers of cases caused by *T. rubrum* and *C. albicans*, which comprised the majority of the infections, are presented separately. (**B**) The remaining fungal species were collectively analyzed with respect to sex and age. (**C**) Proportions of *T. rubrum* and *C. albicans* infections among all positive cases, reflecting their relative frequencies. (**D**) Proportions of *T. rubrum* and *C. albicans* infections among all samples (positive and negative), indicating the probability of infection in clinically suspected cases. Note: In panels (**A**,**C**), the meanings of the colors are as follows: white = male; dark gray = female; light gray = areas of overlap (panel (**C**)).

The most prominent finding from the analysis of the tendencies among other fungal species was that *T. mentagrophytes* and *S. brevicaulis* infected only the toenails, with a particular prevalence of *S. brevicaulis among* subjects older than 45 years (Figure 2).

### 3.3. Predictive Tools for Positive Mycological Culture Results

**Figure 3:**
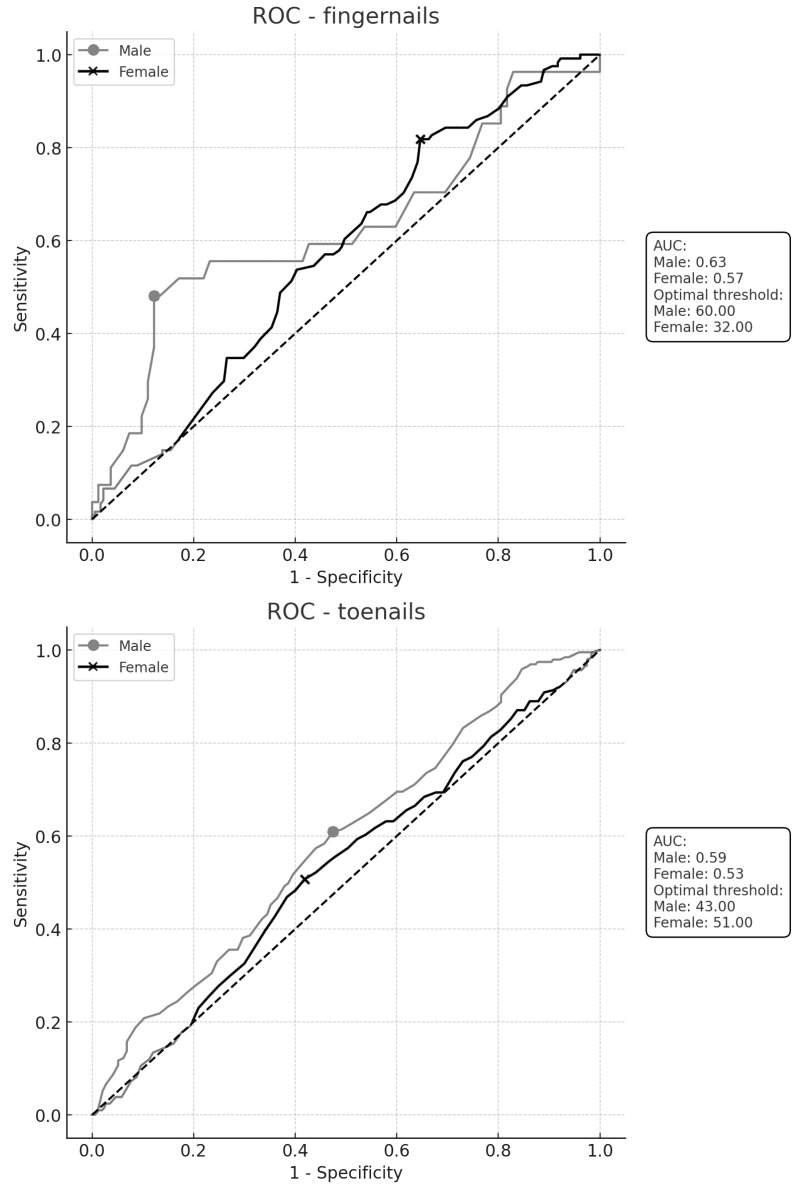
ROC curves illustrating the relationship between subject age (male and female) and the presence of fungal pathogens on the fingernails and toenails. The optimal thresholds were determined using the highest Youden index method. For the fingernails, the true positive rate (TPR) and the true negative ratio (TNR) were 48% and 88% for male subjects and 82% and 35% for female subjects, respectively. For toenails, the TPR and TNR were 61% and 53% for male subjects and 51% and 58% for female subjects, respectively.

First, ROC curves were created for the fingernails and toenails with respect to the two sexes to assess whether the age of the subjects alone could be used to predict positive results from mycological culture. Using the highest Youden index, the optimal cut-off values were determined (32 years for female subjects and 60 years for male subjects for fingernails and 51 years for female subjects and 43 years for male subjects for toenails). Although the values of the area under the curve (AUC) were statistically significant (*p* = 0.005 for toenails and *p* = 0.001 for fingernails), the use of age alone for predictions was not clinically useful due to the low true positive rates (TPRs) (Figure 3).

**Figure 4:**
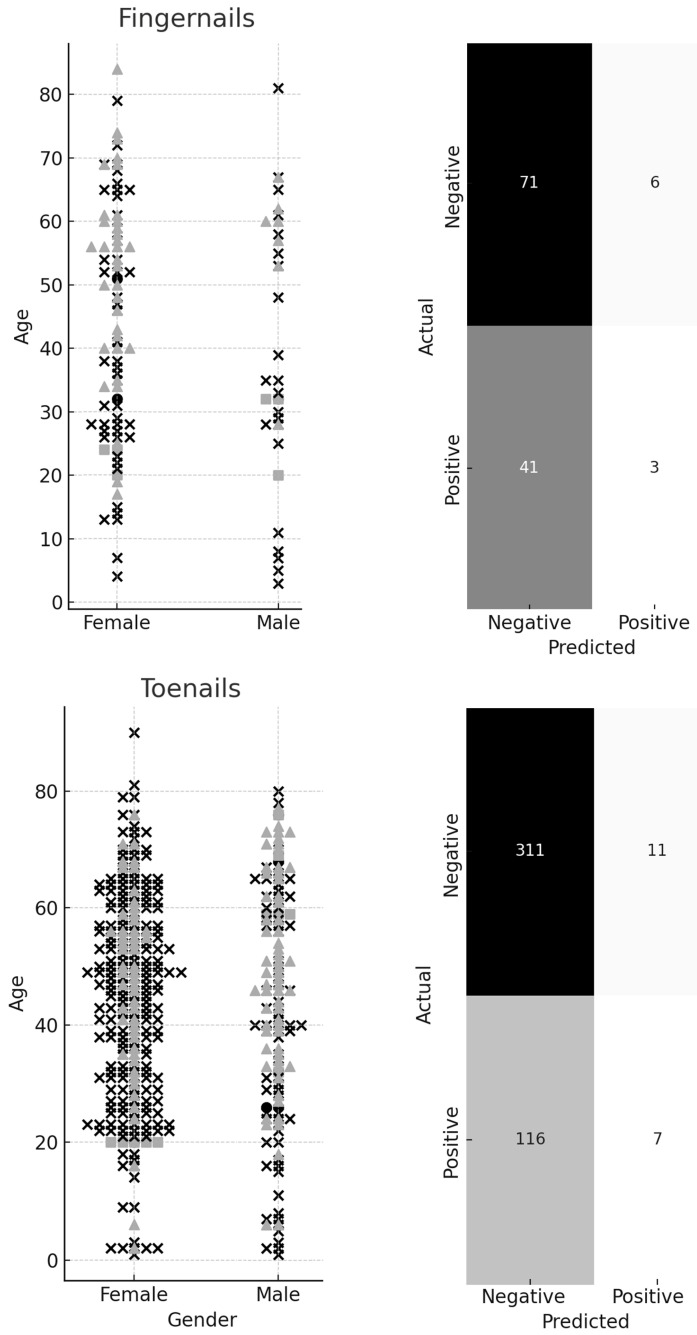
Proposed model for prediction of the presence of fungal infection based on sex and age in a given location with the algorithm of the k-nearest neighbors. The confusion matrices are shown on the right side. For the fingernail, the true positive rate (TPR) was 6.82%, and the true negative rate (TNR) was 92.21%, while for the toenails, the TPR was 5.69%, and the TNR was 96.58%. The meanings of the symbols are as follows: dot = true positive; square = false positive; cross = true negative; triangle = false negative.

Next, a more complex approach was introduced using the KNN method, with cross-validation to optimize the K value. Figure 4 presents the best models with K = 12. However, the models for both fingernails and toenails were unable to detect positive results, displaying extremely low TPRs.

Finally, a set of multivariate stepwise logistic regression models was developed, considering age as either a continuous variable or a dichotomous variable (based on cut-off points determined with ROC curves). All models were comparable according to Nagelkerke’s pseudo-R^2^ and had sufficient goodness of fit. However, they misclassified positive cases as negative. In all models, an older age was associated with a higher probability of onychomycosis (regardless of location). The female sex was associated with a higher probability of fingernail infections, while the male sex had an increased chance of toenail infections (Table 3).

## 4. Discussion

This study provides updated information on the most common etiological agents of onychomycosis, along with a detailed analysis of their associations with the sex and age of the patient. Although we used several predictive methods, we were unable to develop a model with sufficient precision to predict a positive mycological culture in patients with suspected onychomycosis based solely on demographic data.

### 4.1. Relationships Between Site of Infection, Demographics, and Identified Species

Most of the subjects who had mycological nail culture were female; however, most of the results in this group were negative. The male subjects had a higher proportion of positive cultures from toenails, whereas the female subjects had more positive results from fingernails. In particular, in the cases with positive results, the age was statistically significantly higher than in the those with negative cultures.

Sex is one of the factors which substantially influences the odds of onychomycosis, as evidenced by the existing literature. Most epidemiological studies reported a higher prevalence of nail fungal infections in male subjects [8,24]. However, women often prevail in subpopulations actively seeking medical care for nail changes. Among patients assessed for other conditions, the prevalence of onychomycosis was reported to be two times higher in men [25]. This finding is also supported by studies which included control groups, such as the case–control study by Jo Albucker et al., which found that although women represented 54.2% of those with onychomycosis, this proportion was actually lower than that of the control group (55.3%) [26]. In the Irish population, women represented 63% of subjects who had collected samples, but dermatophytic infections were more common in men (25% versus 15%), and yeast infections were less frequent (5% versus 6%) [27].

When examining specific localizations (fingernails versus toenails), this epidemiological pattern becomes more defined.

*T. rubrum* became more prevalent in European and American populations during the 20th century, significantly shaping current epidemiological trends in superficial fungal infections, including onychomycosis. This fungus has a strong predilection for environments created by occlusive footwear and nail injuries, which are more common in men [28]. This seemingly minor factor is underscored by the rarity of tinea pedis and toenail onychomycosis in the regions of origin of *T. rubrum* (for example, Southeast Asia, Indonesia, Northern Australia, and West Africa), where footwear use is historically rare. Other contributing factors include the protective role of estrogen in women and the higher prevalence of diabetes and peripheral vascular disease in men, which also influence the observed patterns [28].

In contrast, *C. albicans* is the predominant etiological agent of fingernail onychomycosis, with a higher prevalence in female subjects. This observation is often attributed to women who engage more frequently in cosmetic procedures involving fingernails and who are more exposed to immersion in water, such as during household activities [29].

Our results followed these global trends. Toenail infections were predominantly caused by *T. rubrum*, while fingernail infections were more commonly associated with *C. albicans*. Among the female subjects, *T. rubrum* infections in toenails and *C. albicans* infections in fingernails showed a single peak incidence at 45–60 years of age. In the remaining cases, a more variable, often bimodal distribution was observed, depending on the ages of the subjects. *S. brevicaulis* exclusively affected the toenails and was observed only in individuals over 45 years of age.

In European studies, *T. rubrum* was identified as the most common etiological factor of onychomycosis, with a prevalence of 65%, while yeasts (mainly *C. albicans*) contributed to approximately 30% of cases [8]. Previous studies by our unit (2001–2006) reported a less distinct difference in the prevalence of toenail onychomycosis between men and women (both accounting for about 50% of the cases). *T. rubrum* and *C. albicans* were the most common causes of toenail and fingernail infections, respectively [30]. Subsequent analyses from Malopolska showed a shift in the nail change site in accordance with the trends observed today [31]. Another Polish study (2011–2016) demonstrated similar patterns, with a predominance of toenail lesions caused by *T. rubrum* (55%) and fingernail infections primarily caused by *C. albicans* (75%) [32].

The prevalence of onychomycosis caused by *T. rubrum* is increasing. For example, in Belgium, the proportion of onychomycosis attributed to this species increased from ~60% to ~85% between 2012 and 2016 [33]. In Germany and Switzerland, *T. rubrum* accounts for 75–85% of cases, with *T. mentagrophytes* as the second most common fungal pathogen (15–25%) [34,35]. Similarly, in Slovakia, *T. rubrum* causes approximately 80% of cases of onychomycosis, followed by *T. interdigitale* (10%) [36]. In the Iranian population, *T. rubrum* and *T. mentagrophytes* are also predominant, with *Epidermophyton floccosum* emerging as a significant cause of onychomycosis (8% of dermatophyte infections in toenails and 24% in fingernails) [37]. Dermatophytes, particularly *T. rubrum*, exhibit a marked tendency to infect the toenails, likely due to the slower growth of the nail plate (reducing fungal clearance) and the warm, humid, and occlusive environment created by footwear [38].

Interestingly, our results also align with those from non-European populations. A study from Tunisia reported a similar proportion of female cases (67%), with a predominance of *T. rubrum* in the toenails and *C. albicans* in the fingernails. However, the proportion of positive cultures in Tunisia was considerably higher (69.6%) than that in our study [38]. Data from China and Nepal also revealed comparable patterns regarding the affected nails, demographics of patients, and etiological factors [39,40].

Onychomycosis caused by *Candida* species is generally considered secondary to prior damage to the nail apparatus, which facilitates fungal invasion, as nondermatophytes lack the ability to efficiently digest keratin. The increased prevalence of fingernail infections in women is often attributed to frequent cosmetic procedures and increased exposure to humidity (for example, during household chores) [41].

Molds were a rare cause of onychomycosis in our study, with frequencies similar to those reported in European data [5]. We observed a few cases of infection with *S. brevicaulis*, a soil-dwelling mold which almost exclusively affects toenails and is more commonly observed in men [42].

### 4.2. Prediction of Positive Mycological Culture Based on Subjects’ Demographics

We initially attempted to predict positive mycological culture results while using age as the sole parameter. However, the optimal cut-off values did not produce clinically useful predictions. One potential explanation for this problem is the presence of a U-shaped relationship between the ages of the subjects and infections with certain fungal species.

Both KNN modeling and logistic regression resulted in extremely high true negative rates (TNRs) but negligible TPRs. The simplest clinical interpretation of this finding is that regardless of the patient’s sex or age, the absence of infection should be assumed until additional diagnostic data (e.g., mycological culture or histology) are available. This supports the paradigm that pharmacological treatment should not be started before confirmatory diagnostic results are obtained. The S1 guidelines clearly state that direct microscopy combined with culture (or molecular diagnostics) should be performed [43]. The need for confirmatory tests comes from the broad differential diagnosis of conditions with a similar morphology, such as nail trauma, lichen planus, or psoriasis. Onychomycosis accounts for up to 50% of all nail dystrophies [44].

Although built logistic regression models cannot be used for strict predictions, they showed that sex increased the odds of a positive culture result from toenails by a factor of approximately 2.5 while decreasing the odds of a positive culture from fingernails by a factor of 0.25–0.50. A higher age (whether analyzed as a continuous or dichotomous variable) independently increased the odds of onychomycosis regardless of location.

It is important to note that although mycological culture supported by direct examination with KOH preparation is often referred to as the “gold standard” for diagnosing onychomycosis, this technique has significant limitations, leading to the development of numerous new approaches to improve diagnostic precision [16]. These include techniques such as dermoscopy and histological examination (using periodic acid-Schiff (PAS] or Grocott’s methenamine silver (GMS) staining), advanced imaging methods (e.g., ultraviolet fluorescence excitation imaging, confocal microscopy, and optical coherence tomography), and molecular techniques like PCR, matrix-assisted laser desorption ionization–time of flight (MALDI-TOF), and Raman spectroscopy.

Despite its established role, mycological culture does not meet the criteria of a true “gold standard”, which usually implies both high sensitivity and specificity [45]. Therefore, the findings of our study, based on the results from mycological culture supported by direct microscopic examination, may be subject to bias.

An optimal solution for addressing these limitations could involve the use of multiple diagnostic techniques in future studies, ideally combining culture with visual and molecular methods or adopting a stepped diagnostic approach. Some authors recommend combining the histological examination of PAS-stained nail clippings with culture or KOH preparation, reporting sensitivities of up to 95% [17,18].

Nevertheless, the overall structure of the data from our group does not deviate from the global norm. Hence, despite there certainly being some false negative results in our analysis, there should be at least a considerable correlation between the results of our analysis and the true relationships.

Efforts are ongoing to make the diagnostic workup for onychomycosis faster and more feasible. Various approaches have been implemented using not only routine diagnostic tools but also image analysis incorporation based on convolutional neural networks and other methods of artificial intelligence (AI) [46]. In particular, when trained with a proper dataset, AI achieves a diagnostic accuracy comparable to that of experienced dermatologists, suggesting the existence of a limit to the extent to which visual assessment of nail lesions alone can guide diagnosis. Dermoscopy is considered a useful aid in the diagnostic process but lacks sufficient precision to replace mycological culture [47].

Histological examination with PAS staining yields the highest diagnostic precision but lacks the ability to effectively differentiate between fungal species [48]. Molecular methods, while relatively rapid to perform, are costly, prone to false positive results, and unable to confirm fungal pathogenicity [49]. Studies have reported a 2–4-fold increase in positive PCR results compared with mycological culture [46,50]. The results of the works using simultaneous PCR and histological examination suggest that this difference may be attributed to real positive results and a general improvement in diagnostic accuracy [51]. An additional advantage of these techniques lies in their ability to provide reliable results even in patients who have undergone antifungal treatment before examination [52]. Furthermore, molecular methods have been shown to be useful for monitoring the effectiveness of treatment [53].

Some studies have proposed redefining the concept of a diagnostic “gold standard” by combining molecular techniques with classical methods, with the best results achieved through a combination of histology and PAS staining [54,55]. This approach is particularly valuable for diagnosing infections caused by non-dermatophyte molds, as their identification often requires multiple sequential nail samples. This process can lead to challenges such as poor patient compliance and delayed diagnosis, which these combined methodologies can help mitigate [56].

Overcoming diagnostic challenges is critical, as many patients with onychomycosis do not receive necessary pharmacological treatment [57]. On the contrary, there is a concerning trend of initiating treatment without diagnostic confirmation, which can lead to unnecessary complications, delays in appropriate diagnostics, and increased antifungal resistance [58]. For toenail onychomycosis, a reasonable approach before obtaining diagnostic test results is to advise patients on proper sanitization methods for socks and shoes [59].

### 4.3. Study Limitations

This study has several limitations. It is a retrospective analysis of an anonymized archival database. We lacked data on the clinical presentation of nail changes, the course of treatment, and information about other coexisting symptoms of fungal infection. This was a single-center study, which inherently limits external validity; however, as we demonstrated, the results are comparable with those of other studies.

Although mycological culture is frequently referred to as the diagnostic “gold standard”, the high potential for false negative results may have influenced the findings of this analysis, and future studies should consider a prospective combination of molecular testing and some classical methods or a structured step approach. Ultimately, the high yield of negative results predicted by all of our models might have been derived from just the excess of culture false negative results.

Furthermore, the predictive models developed in this study were based solely on demographic and clinical parameters. There remains the potential for optimization through the incorporation of additional data, such as the results of dermoscopic examination.

## 5. Conclusions

This study highlighted current trends in the incidence of onychomycosis caused by different fungi and their associations with the anatomical site of infection, as well as patient sex and age.

Furthermore, the predictive models we constructed suggest that clinical presentation and patient demographics alone are insufficient to anticipate the presence of onychomycosis. Therefore, the dominant paradigm of requiring confirmatory diagnostic testing before starting treatment remains valid. The approach we proposed can be further refined, for example, by incorporating dermoscopy results, other noninvasive diagnostic techniques, and also histology or molecular testing.

In a broader context, these findings underscore the urgent need to integrate advanced diagnostic tools and multidisciplinary approaches to improve the precision and efficiency of onychomycosis diagnosis, ultimately improving patient outcomes.

## Figures and Tables

**Table 1:** Overall characteristics of the study population.

Characteristic		Overall (*N* = 2722)
Age (years; median [Q1–Q3])		46 (32–59)
Sex (*N*, %)	male	859 (32%)
	female	1863 (68%)
Location (*N*, %)	fingernails	624 (23%)
	toenails	2098 (77%)
Mycological culture (*N*, %)	negative	1951 (72%)
	positive	771 (28%)
Species identified in positive mycological culture (*N*, %)	*T. rubrum*	487 (63%)
	*C. albicans*	202 (26%)
	*C. non-albicans*	26 (3%)
	*Rhodotorula*	21 (3%)
	*S. brevicaulis*	19 (2%)
	*T. mentagrophytes*	12 (2%)
	*A. niger*	2 (<1%)
	*Malassezia*	1 (<1%)
	*M. canis*	1 (<1%)

Abbreviations: Q1–Q3 = interquartile range.

**Table 2:** Characteristics of four subpopulations based on the site of suspected fungal infection and the results of mycological culture.

Characteristic		Negative in Fingernails (*N* = 406)	Positive in Fingernails (*N* = 218)	*p* Value	Negative in Toenails (*N* = 1545)	Positive in Toenails (*N* = 553)	*p* Value
Age (years; mean [Q1–Q3])		40 (27–55)	50 (32–60)	0.006	45 (32–58)	49 (34–61)	0.001
Sex	female	283	173	0.01	1113	294	<0.001
	male	123	45		432	259	

**Table 3:** Logistic regression models which predict positive mycological culture results based on subjects’ sex and age.

		Fingernails—Model 1	
Characteristic		OR (95%CI)	*p* Value
sex	female male	reference 0.52 (0.32–0.86)	0.01
age (per 1 year)		1.02 (1.01–1.03)	0.001
		Fingernails—model 2	
Characteristic		OR (95%CI)	*p* Value
sex	female male	reference 0.73 (0.42–1.27)	0.3
age (dichotomous)	below threshold above threshold	reference 2.21 (1.38–3.54)	<0.001
		Toenails—model 1	
Characteristic		OR (95%CI)	*p* Value
sex	female male	reference 2.55 (2.01–3.24)	<0.001
age (per 1 year)		1.01 (1.00–1.02)	0.001
Toenails—model 2			
Characteristic		OR (95%CI)	*p* Value
sex	female male	reference 2.42 (1.91–3.07)	<0.001
age (dichotomous)	below threshold above threshold	Reference 1.50 (1.19–1.90)	<0.001

Fingernails—model 1: Nagelkerke pseudo-R^2^ = 0.063, Hosmer–Lemeshow *p* = 0.053, true negative rate = 93.9%, true positive rate = 9.5%. Fingernails—model 2: Nagelkerke pseudo-R^2^ = 0.064, Hosmer–Lemeshow *p* = 0.369, true negative rate = 100%, true positive rate = 0%. Toenails—model 1: Nagelkerke pseudo-R^2^ = 0.065, Hosmer–Lemeshow *p* = 0.121, true negative rate = 100%, true positive rate = 0%. Toenails—model 2: Nagelkerke pseudo-R^2^ = 0.066, Hosmer–Lemeshow *p* = 0.777, true negative rate = 100%, true positive rate = 0%. Abbreviations: OR = odds ratio; 95%CI = 95% confidence interval.

## Data Availability

The raw data supporting the conclusions of this article will be made available by the authors on request.

## Abbreviations

The following abbreviations are used in this manuscript:

AI	Artificial intelligence
AUC	Area under the curve
CI	Confidence interval
DMSO	Dimethyl sulfoxide
GMS	Grocott’s methenamine silver
KNN	K-nearest neighbors
MALDI-TOF	Matrix-assisted laser desorption ionization time of flight
OR	Odds ratio
PAS	Periodic acid–Schiff
PCR	Polymerase chain reaction
QoL	Quality of life
ROC	Receiver operating characteristic
TNR	True negative rate
TPR	True positive rate
